# Data Access and Usage Practices Across a Cohort of Researchers at a Large Tertiary Pediatric Hospital: Qualitative Survey Study

**DOI:** 10.2196/medinform.8724

**Published:** 2018-05-14

**Authors:** Hoi Ki Kiki Ho, Matthias Görges, Elodie Portales-Casamar

**Affiliations:** ^1^ BC Children's Hospital Research Institute Vancouver, BC Canada; ^2^ Department of Anesthesiology, Pharmacology & Therapeutics University of British Columbia Vancouver, BC Canada; ^3^ Department of Pediatrics University of British Columbia Vancouver, BC Canada

**Keywords:** clinical data sharing, research barriers, data linkage, data sources, data management, environmental scan, research facilitation

## Abstract

**Background:**

Health and health-related data collected as part of clinical care is a foundational component of quality improvement and research. While the importance of these data is widely recognized, there are many challenges faced by researchers attempting to use such data. It is crucial to acknowledge and identify barriers to improve data sharing and access practices and ultimately optimize research capacity.

**Objective:**

To better understand the current state, explore opportunities, and identify barriers, an environmental scan of investigators at BC Children’s Hospital Research Institute (BCCHR) was conducted to elucidate current local practices around data access and usage.

**Methods:**

The Clinical and Community Data, Analytics and Informatics group at BCCHR comprises over 40 investigators with diverse expertise and interest in data who share a common goal of facilitating data collection, usage, and access across the community. Semistructured interviews with 35 of these researchers were conducted, and data were summarized qualitatively. A total impact score, considering both frequency with which a problem occurs and the impact of the problem, was calculated for each item to prioritize and rank barriers.

**Results:**

Three main themes for barriers emerged: the lengthy turnaround time before data access (18/35, 51%), inconsistent and opaque data access processes (16/35, 46%), and the inability to link data (15/35, 43%) effectively. Less frequent themes included quality and usability of data, ethics and privacy review barriers, lack of awareness of data sources, and efforts required duplicating data extraction and linkage. The two main opportunities for improvement were data access facilitation (14/32, 44%) and migration toward a single data platform (10/32, 31%).

**Conclusions:**

By identifying the current state and needs of the data community onsite, this study enables us to focus our resources on combating the challenges having the greatest impact on researchers. The current state parallels that of the national landscape. By ensuring protection of privacy while achieving efficient data access, research institutions will be able to maximize their research capacity, a crucial step towards achieving the ultimate and shared goal between all stakeholders—to better health outcomes.

## Introduction

The use of data is a foundational component of both research and health care. Health and health-related data are generated at high volumes and are not limited to front-end clinical data [1]. Secondary sources of data include medical imaging, laboratory, insurance, and demographic data, and particularly patient-collected data like activity, nutrition, and other qualitative data; these data add substantial information to the mass of overall health-related data [1]. These complex and interconnected datasets are commonly referred to as “big data”, which is often formally defined as large and complex datasets that require specialized software for manipulation and analysis [1,2]. “Big data” is projected to grow at an accelerated pace; for example, the size of health and health-related data in the United States is expected to reach the scale of yottabytes (10^24^ gigabytes) soon [1]. This rapid expansion of health care data is recognized globally, and the ability to access and analyze this wealth of information might allow us to better support a wide range of medical and health care functions, like public health surveillance, population health management, and real-time clinical decision support [1-6].

As research takes on an increasingly data intensive and global focus, there is an increased need for appropriate data sharing, storage and maintenance infrastructure at research institutes engaging in big data analytics [6-12]. There are many benefits of data sharing: it allows for replication and validation of scientific outcomes and results, projects can be extended and viewed from different perspectives, and data re-collection can be minimized [6,8,9]. Infrastructure that supports data sharing, along with the appropriate storage and maintenance of data, maximizes its value and contribution to research [6,7,9,10].

In a study conducted by the Publishing Research Consortium in 2010, approximately two-thirds of the 3823 respondents identified access to datasets, data models and algorithms, and programs as being important to very important, but only about a third of them perceived these resources to be easily accessible [8]. A subsequent survey administered by Tenopir et al [7] in 2014 around perceptions and practices pertaining to data sharing revealed that 85% of 1329 participating scientists would be interested in datasets generated by other researchers or institutions if they were easily accessible. Additionally, 67% viewed the lack of access to these datasets as an impediment to scientific progress, while less than half reported being satisfied with the integration of data from other sources or the availability of different types of data to answer research questions [7].

While there is consensus that data sharing is an integral part of scientific research, there are barriers that contribute to the disparity between the desire to share data and the perceived accessibility of data [2,3,6-10]. Logistical barriers to developing standardized data sharing systems or processes, or a centralized repository for data sharing is a shared challenge among research institutions [1-5,9-12]. For example, to consolidate disparate data sources, datasets must be generated in an “analysis-ready format.” This poses several methodological challenges: data harmonization is complicated by the heterogeneity of data sources (the types of data collected and the mechanisms used to collect them) and the availability and usability of data hosted in current electronic health records systems [4,5,6,10,11]. Further, other concerns with data access and sharing, common across research institutions, are confidentiality of potentially re-identifiable data and ethical concerns around consent—has it been given and does it extend to data usage by other parties [1-6]?

In Canada, while health care systems and innovation are highly valued, researchers have faced challenges with striking a balance between enabling timely access to data for research purposes and protecting patient confidentiality [2,3,10]. A major barrier is the inconsistent interpretation of privacy legislation, which varies by province and has led to varying requirements for research ethics board approval, privacy impact assessments, and related data access processes, with turnaround times ranging from months to years [10].

Challenges and concerns around data access are especially pertinent to investigators at BC Children’s Hospital Research Institute (BCCHRI), where discovery, translational, and clinical research is conducted to benefit the health of children and their families; at this center, many collaborations are national or global. Many frameworks identify big data through three dimensions: volume, variety, and velocity [13]. Much of the research work conducted at BCCHRI fits under one or more of these dimensions, as our hospital site sees over 200,000 patients annually, from which it collects a large volume of varied data from patients consenting to participate in local studies [14]. These data include clinical parameters and notes, questionnaire responses, medical imaging data, high-density vital sign recordings, multi-omics datasets, and many more. These data are collected in real-time, creating and contributing to various databases, databanks and registries. Specifically, the Clinical and Community Data, Analytics and Informatics Group (data group) engages in such work. Within the research institute’s “Evidence-to-Innovation” theme, this group is composed of over 40 BCCHRI investigators with diverse expertise and interest in data who share a common goal of facilitating data collection, usage, and access across our community. Researchers on site have experienced increasing challenges with accessing data for research. Thus, a local environmental scan was performed to a) evaluate and review the state of the data access infrastructure at BCCHRI; b) identify barriers and opportunities; and c) provide feedback to the institute’s leadership to help improve data access and usage on site.

## Methods

This environmental scan was a quality improvement activity. The University of British Columbia and Children’s and Women’s Health Centre of British Columbia Research Ethics Board does not review quality assurance or quality improvement studies, in accordance with Article 2.5 of the Tri-Council Policy Statement 2. Following standard methodology for qualitative research [8], semistructured one-to-one interviews were conducted between May and August 2016, with members of the data group, focusing on both their data needs and their experiences accessing and using data on the BC Children’s Hospital (BCCH) campus. With consent from the interviewee, interviews were tape-recorded for transcription of notes, at which point the recordings were destroyed. Each respondent was assigned a participant code (P#). A full list of interview questions can be found in Multimedia Appendix 1, and the list of datasets provided to participants (referenced in Question 1) can be found in Multimedia Appendix 2.

Quantifiable metrics from multiple choice questions, like data needs and expertise, were gathered using paper questionnaires and summarized using Excel (Microsoft, Redmond, WA). The unstructured descriptions of individual experiences with data access and usage were analyzed and synthesized using a template analysis approach [15]. The initial template was defined a priori with three parent themes (barriers for data access, facilitators, and opportunities). The final template used to code and analyze all interview data can be found in Multimedia Appendix 3, which includes additional sub-themes to further describe the three parent themes in the initial template. Relevant quotes were extracted from the interview transcripts to further illustrate respondents’ experience with data access and usage, which is a common means of textual data presentation in the template analysis approach. Each quote is attributed to the corresponding respondent using their participant code.

To prioritize and rank barrier items, a total impact score was calculated. This score is analogous to the severity ratings proposed in Jakob Nielsen’s usability methodology [16], where a composite score is derived from both the frequency with which a problem occurs and the impact of the problem. For the purposes of this scan, we used the following terminology: *total impact score* for each barrier = *frequency* of mention x *mean effect score* across all items tagged under this barrier. The effect score for each item ranged from 1 (minimal) to 3 (severe) based on the participant’s description of how much it affected their research.

## Results

Thirty-five of the 43 data group members participated in the environmental scan, constituting an 81% response rate.

### Participant Characteristics

Expertise in the data group represents a wide range of specialties, with respondents of the scan mainly identifying clinical data (22/35, 63%) as their “core” expertise, followed by data analysis (13/35, 37%), data standardization and harmonization (11/35, 31%), and administrative data (11/35, 31%; Table 1). Further, when asked if any further expertise was required to advance their work, respondents reported needing additional support in statistics (18/35, 51%) and navigating data access processes (17/35, 49%).

### Data Needs

When asked to identify their current data needs, most respondents identified improved access and facilitated data linkage as important data needs (20/35, 57%), followed by the need to bridge clinical and research data (18/35, 51%) and improved usability of electronic health records data (14/35, 40%; Table 2).

### Barriers

**Figure 1:**
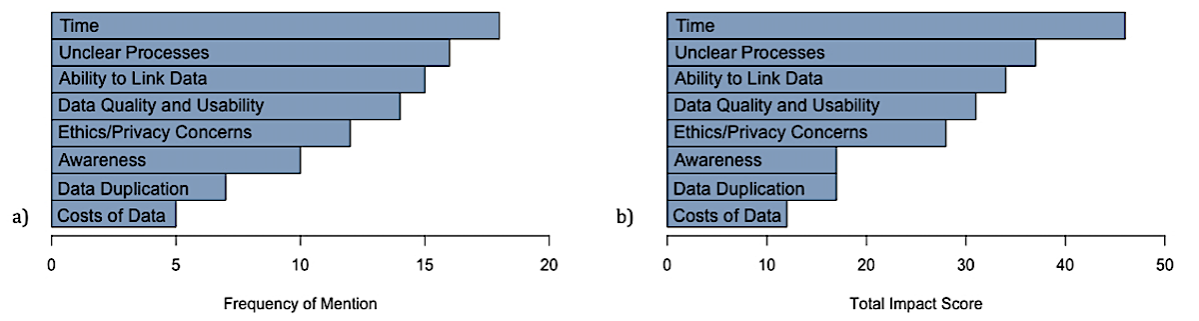
Frequency of mention (a) and total impact score (b) of the barriers on respondents’ research. The barriers that have the most substantial impact on respondents’ research are lengthy turnaround times, inconsistent and unclear data processes, and limited capacity for data linkage.

The three greatest challenges to accessing and using data for research were lengthy turnaround times (18/35, 51%), inconsistent and opaque data access processes (16/35, 46%), and the inability to link data (15/35, 43%; Figure 1, see part a)*.* All barriers were ranked using their total impact score and analyzed in detail (Figure 1, see part b).

**Table 1:** Collective expertise of data group members. The data group hosts a wide breadth of expertise with clinical data being most prevalent, followed by data analysis and administrative data. This table lists all categories selected by 5 or more respondents. Other categories with <5 respondents included: clinical expertise (4, 11%), data linkage (4, 11%), database design and building (4, 11%), mobile apps (4, 11%), data integration across modalities, (3, 9%) experience with data stewards (3, 9%), population level data (2, 6%), child pyschology (1, 3%), health surveillance (1, 3%), intervention design (1, 3%), machine learning (1, 3%), and privacy and security (1, 3%).

Identified expertise	n (%)
Clinical data	22 (63)
Data analysis	13 (37)
Administrative data	11 (31)
Data standardization/harmoinzation	11 (31)
Registry/database	10 (29)
Biostatistics	8 (23)
National data networks	8 (23)
Data visualization	7 (20)
Genomics data	7 (20)
Data mining	6 (17)
Epidemiology	5 (14)
International data networks	5 (14)

**Table 2:** Identified data needs. Facilitated data linkage, improved data access and bridging clinical and research data were the three most frequently mentioned data needs.

Data needs	n (%)
Facilitated data linkage	20 (57)
Improved data access	20 (57)
Bridging clinical and research data	18 (51)
Improved quality of e-records	14 (40)
Access to expertise	10 (29)
Data governance	10 (29)
Permission to contact	5 (14)
Registry framework	3 (9)
Storage space	1 (3)

#### Time

The “Time” barrier was mentioned by 18/35 (51%) respondents, with a mean effect score of 2.56 (median 3; range=2-3), and a total impact score of 46. Most respondents identified the long turnaround time for processing and completing data requests as one of the greatest challenges when trying to access data outside of their primary collection, with instances of waiting up to 7 years to receive datasets and multiple rounds of back and forth communication with different data custodians. Some respondents report waiting for several years without the data request ever reaching completion or receiving approval:

I didn't want to be limited to local data and I wanted to be able to compare the patient population coming through BC Children's Hospital and provincial level data. I put members on my team on the task, but it never progressed anywhere. The process was too difficult and took too long: it was taking years. Eventually, we just gave up and moved on to other things that took priority.

P23

#### Unclear Processes

The “Unclear Processes” barrier was mentioned by 16/35 (46%) respondents, with a mean effect score of 2.31 (median 2; range=2-3), and a total impact score of 37. Issues were largely related to 1) lack of a central resource, and 2) a lack of consistency and standardization across different data custodians about access procedures. Lacking a central data access resource leaves researchers without guidance on how to approach accessing data outside their primary collection; that is, being unaware of who to contact, what the data access processes entail, and what data is available:

It’s unclear as to who will make the decisions, who will provide the approval, who will review the paperwork and look at the privacy impact. This needs to be cleared up and formalized and communicated so that it’s clear who to talk to in order to get access to this data, and we need someone to facilitate this.

P10

Furthermore, the data access processes are often inconsistent and unclear. Respondents noted that the data access processes are highly variable, especially between different data custodians. Researchers feel as though each time a new project is started, they are starting from scratch and responses emphasized the need to streamline these processes.

#### Ability to Link Data

The “Ability to Link Data” barrier was mentioned by 15/35 (43%) respondents, with a mean effect score of 2.27 (median 2; range=1-3), and a total impact score of 34. A common concern with many researchers is that the current data infrastructure encourages the creation of “silos,” in which data exists isolated within certain divisions, or is restricted to certain projects. Respondents note a lack of official guidance or established infrastructure to facilitate data linkage between disciplines or between internal and external data sources:

If you look at other leading children’s hospitals around the world, there are mechanisms by which patients and families donate their data and information for research purposes in very broad and powerful ways, and in the Canadian environment, that’s more challenging in terms of how we handle data privacy and at the level of the Stanford’s and Hopkins’, one really needs to have a mechanism by which patients are able to donate or release their data for research and my experience in the BC Children’s environment, most patients are actually shocked when they find out we’re not using their data.

P30

#### Data Quality and Usability

The “Data Quality and Usability” barrier was mentioned by 14/35 (40%) respondents, with a mean effect score of 2.21 (median 2; range=1-3), and a total impact score of 31. Internally, the current state of electronic health records poses a challenge for researchers, as the data are not truly electronic, such that data is not stored in an electronically extractable format. Thus, manual transcription is still required to extract the data, with the possibility of transcription error. This greatly limits the campus’ ability to contribute to and participate in larger national and international databases. Externally, many variables requested by researchers are unavailable, not defined clearly, or in an inappropriate format, thus requiring further back and forth communication between researchers and data custodians:

The data received from the steward is messy, as in it isn’t organized in a common-sense way. I couldn’t tell which participant answered which question, which then required a constant regeneration of the dataset to have it organized in a meaningful way. Unfortunately, I can’t avoid this because investigators themselves do not have access to the raw data, so I couldn’t even match the data up or re-organize the data on my own and had to engage in this constant back and forth with the steward.

P2

#### Ethics and Privacy Concerns

The “Ethics and Privacy Concerns” barrier was mentioned by 12/35 (34%) respondents, with a mean effect score of 2.33 (median 2; range=2-3), and a total impact score of 28. Some examples of these obstacles include not having permission to contact patients and their families, the lack of consistency as to when and if a Privacy Impact Assessment (PIA) is required for a project, and the varying requirements across ethics boards for multi-site projects.

The unknown variable and obstacle is that it is unclear when and for which projects the PIA is required, the process has been very inconsistent, with a lot of back and forth, often asking for information that has already been provided. I find the PIA processes inconsistent not only across health authorities but even within health authorities.

P26

#### Awareness

The “Awareness” barrier was mentioned by 10/35 (29%) respondents, with a mean effect score of 1.70 (median 1.5; range=1-3), and a total impact score of 17. Responses show that there are some researchers who are completely unaware of the data sources that are available to them outside their primary collection or collaborations with others:

I haven’t heard of or used any of the sources listed here, so I haven’t had any experience with these data holders as I didn’t know that these sources existed. I’ve only used data through my own primary collection, but I would like to learn more about how to access these and what types of data is available.

P18

#### Data Duplication

The “Data Duplication” barrier was mentioned by 7/35 (20%) respondents, with a mean effect score of 2.43 (median 2; range=2-3), and a total impact score of 17. The manual transcription required to extract data from local systems, and the inability to link datasets across different projects and studies, leads to the continued duplication of data. Respondents noted that many studies collect the same basic package of information (eg, demographics), which further contributes to repeated and isolated datasets existing across the campus:

What I find happens a lot here is that there’s duplication in data collection, and if we had a way to collect a base level of data on all the kids coming to the hospital, like a standardize form, especially to make it easier to be integrated into electronic health records and pulled, I think that would really save time as opposed to every time there’s a new project, you pull the same data and some poor med student is manually extracting it. There could be errors there, if we could somehow connect it via a system with accurate and secure information that would be extractable, that would be great. I know there’s lots of red tape around this, in the sense we can’t even get such a system running, let alone use it for research, but I think ultimately that’s what we need.

P32

#### Costs of Data

The “Costs of Data” barrier was mentioned by 5/35 (14%) respondents, with a mean effect score of 2.40 (median 2; range=2-3), and a total impact score of 12. Data requests are often associated with significant costs, and acquiring funding continues to be difficult for many researchers, especially when the data requests are often onerous and funding is typically provided only for a limited time span.

### Facilitators

Some facilitators in navigating these challenges were identified by 17/35 (49%) respondents. Existing rapport with key contacts from data sources is a major facilitator to the success that some researchers have had (9/17, 53%). Although this has proven beneficial for those who had these existing networks, it does represent a barrier to those without them. Researchers also note that they will rely on primary collection or use publicly available data when possible (5/17, 29%). However, using data sources with clearly outlined data access processes and existing infrastructure to support their data requests (eg, Population Data BC) is a facilitator for those who do attempt to access external datasets (3/17, 18%).

### Opportunities

Opportunities for the data group were identified by 32/35 (91%) respondents. The following categories emerged: data access facilitation (14/32, 44%) and migration toward a single data platform (10/32, 31%).

#### Data Access Facilitation

It was suggested that a support unit or a central resource dedicated to data access would be highly beneficial as a centralized and focused support system does not seem to currently be in place. The hope is for the potential team to facilitate the entire data access process, from consultation to support with data request logistics (eg, data request forms).

#### Single Data Platform

Respondents would like to explore the opportunity of developing a single platform where existing data could be linked, and new data can be entered through single point of entry. It would have infrastructure built to collect a set of standardized variables from all patients and the capacity to be adapted for specific projects. This would limit data duplication through different prospective studies collecting the same variables. The possibility was also mentioned of having a patient portal in such a system to allow patients to contribute data on their own accord.

### Data Sources and Management Tools

With respect to data sources, five respondents had no previous experience accessing external datasets. Other participants most frequently accessed datasets from the hospital clinical data warehouse (13/30, 43%), through Population Data BC (popData) [17] (9/30, 30%), or the Canadian Institute for Health Information [18] (7/30, 23%). Other datasets used include BC Perinatal Data Registry [19], BC Children’s Hospital Biobank [20], Canadian Neonatal Network [21], Canadian Neonatal Follow-Up Network [22], or Edudata [23].

The most commonly used statistical and computing tools were SPSS [24] (20/35, 57%), and R [25] (19/35, 54%), others including SAS [26], STATA [27], MATLAB [28], and Python [29]. The most commonly used data management tools were REDCap [30] (28/35, 80%) and MS-Excel [31] (26/35, 74%), with additional tools with low usage (MS Access [32], custom databases, Dropbox [33], Dacima [34], and various survey tools).

## Discussion

### Principal Findings

Timely access to health and health-related data is crucial to advancing health care systems and stimulating innovation to improve quality of care [1-10]. BCCHRI houses a wide breadth of topics and relies on many different data sources. The most critical data needs identified by respondents, like improved access and facilitated data linkage, directly reflect the challenges currently faced; for example, the lengthy turnaround time and the opaque and highly variable data access processes. These factors are detrimental to current research endeavors, and often result in researchers refraining from using existing data, but rather collecting it again through a prospective study.

A need to create resources to facilitate and support data access and ultimately to move towards a single data platform that will allow comprehensive, linked, clean and processed clinical data, not isolated by discipline or disease, is strongly evident from this scan. An increased capacity for data linkage also improves the site’s ability to participate in and contribute to national and international projects and registries. Furthermore, there is an apparent lack of awareness of the datasets available, and how to gain access to them. Most researchers will use the bigger, more centralized resources such as the hospital clinical data warehouse or popData, which have better defined processes and points of contact, rather than the smaller isolated dataset with no clear shop front. This challenge of having documentation for such processes and methods to gather and link disparate sources of data are echoed in the literature [2,3,6,9,10]. This highlights the need for a centralized source of information, which could take the form of a repository or a data navigator role, to connect researchers with these isolated datasets, thereby enhancing their utilization and maximizing the value of the data.

REDCap usage is prevalent, probably due to its ease of accessibility, not only at BCCH, but also at many different sites across Canada (allows for easy collaboration), the ease of Research Ethics Board approval for its use, and low cost. The use of these tools is essential to streamline and standardize data management and analysis practices. They emphasize how critical it is to have support systems broadly available to our community and to have central access to, and support for, specialized statistical software such as SPSS and R.

These opportunities are real and would bring great benefit to both researchers and patients by increasing the value of the data they contribute [1-12]. However, there are logistical and administrative challenges that are difficult to overcome [2,4,5,6,9,10,12]. The interpretation of the privacy legislation mandating data access mechanisms is at the discretion of each individual data custodian and steward and can be hard to harmonize. This is consistent with other reports that note strong variation in the interpretation of privacy legislation, which lead to variable data access processes and inconsistencies in access time [2,3,6,7,9,10].

Furthermore, data governance needs to be clearly established, particularly when applying data linkage and integration between existing data sources, to define clear rules and oversight for the data access platforms and mechanisms. This is consistent with the findings from the 2015 Accessing Health and Health-Related Data in Canada report, which cited strong and clear governance models, a willingness to enable appropriate use of data, recognizing that risk cannot always be completely eliminated, and establishing explicit guidelines for privacy risk assessment as principles for success at “best practice” institutions [5]. While obstacles do exist, creating a system that allows for timely data access while simultaneously protecting and respecting confidentiality is feasible and has been demonstrated in “best practices” entities such as the Farr Institute in Scotland and the Wales Secure Anonymized Information Linkage Databank [10]. We can learn from these institutions’ successes in mitigating barriers to data access now that needs in this area have been identified and prioritized.

### Limitations of the Environmental Scan

Limitations to our study include the small sample size, as it was conducted at a single center and only 35 researchers of the entire research community participated, which limits generalizability. However, since the data group was formed as an open forum that any BCCH researcher could join when their research includes a strong data component, we believe that our respondent sample includes most of the knowledge and expertise related to data usage and access in our community. This in-depth work, although at the level of a single institution, has implications far beyond it, as the patient population that passes through BCCH is representative of patients across the entire province, and BCCH is a prominent partner in many national initiatives and international data networks to improve research in health care. This allows the results of this study to propagate beyond this institution alone. Also, based on the supporting literature, these themes are common among many institutions globally. This work represents a systematic way of identifying and prioritizing barriers and opportunities to data access and usage, which can be shared and reflected upon among different provinces and health authorities. As such, this work has played a part in motivating the changes made to privacy review processes at the Provincial Health Services Authority (PHSA), which introduced a new Privacy Advisor position that works directly with PHSA researchers and staff to identify privacy and security risks. This new role is intended to streamline the privacy review process while also ensuring that research conducted in PHSA institutions is carefully reviewed for privacy considerations. Environmental scans, such as ours, can demonstrate impact, which lies in policy and governance changes, as well as communicating these challenges, best practices and potential solutions among the research community.

In addition, as interviews were semistructured, a variable amount of data was captured for each participant. For example, the responses to open ended questions regarding barriers, facilitators and opportunities yielded varying levels of detail from each respondent. Additionally, participants’ selection options changed as the scan progressed, as the lists provided to them grew during data collection. To prioritize and rank barrier items, we used a total impact score, which is derived from both the frequency of mention of a problem and the effect of the problem. While the frequency is objectively measured, the effect is determined by the interviewer based on the interviewee’s comments. We note that both trends are similar despite a slight exaggeration of the Time and Awareness barriers, which shows that even though the effect is subjectively measured, it doesn’t influence the total impact score considerably (Figure 1). Furthermore, for some metrics, only a subset of participants was able to contribute; for example, only those with previous experience requesting a dataset from a custodian would be able to contribute to the question related to previous data sources used.

### Conclusion

In an era of increasing digitization of information and globalization, the demand and need for health and health-related data will continue to grow. By identifying the current state and needs of the data community onsite, this study enables us to focus our resources on combating the challenges having the greatest impact on researchers. The current state of BCCHRI parallels that of the national landscape, and by looking towards organizations that have been able to ensure protection of privacy while achieving efficient data access, the institute will be able to maximize their research capacity. Solutions do exist and acknowledging problem areas and taking action is the first step towards achieving the ultimate and shared goal between all stakeholders—to better health outcomes.

## Appendix

Multimedia Appendix 1 Environmental scan interview questions.

Multimedia Appendix 2 List of available datasets provided to investigators.

Multimedia Appendix 3 Final analysis template.

## Abbreviations

BCCHRI: BC Children’s Hospital Research Institute
BCCH: BC Children’s Hospital
PHSA: Provincial Health Services Authority
PIA: Privacy Impact Assessment
