# Supplementary material for: Data Access and Usage Practices Across a Cohort of Researchers at a Large Tertiary Pediatric Hospital: Qualitative Survey Study
Source: JMIR Med Inform. 2018 May 14;6(2):e32. doi: 10.2196/medinform.8724 (PMC5972187; doi:10.2196/medinform.8724)
Supplement: Multimedia Appendix 1 [file medinform_v6i2e32_app1.pdf]

Appendix A - Environmental Scan Interview Questions

1. What datasets have you used?
   - Refer to list of datasets available (Appendix item II)
2. Any particular dataset or data you hope to have access to in the future?
3. What expertise do you have?
   - Possible answers:
     - Registry
     - Administrative Data
     - Clinical Data
     - Genomics Data
     - Data linkage
     - Data mining
     - Data standardization/harmonization
     - Biostatistics
     - Data visualization
     - Data analysis
     - Data integration across modalities
     - National data networks
     - International data networks
     - Other: ________________
4. Is there a specific person of a particular expertise that you feel is needed?
5. What are your current data needs?
   - Possible answers:
     - Data governance
     - Access to expertise
     - Facilitate data linkage
     - Improved data access
     - Registry framework
     - Permission to contact
     - Bridging clinical and research data
     - Improved quality of e-health records
     - Other, please specify: ________________________
6. Could you share your experiences accessing and using data? Describe any challenges or successes.
7. Do you use any public data repositories? (research generated dataset on public repository)
8. What statistical and/or computing tools do you use?
9. Do you use any data access/management tools?
10. If there were one opportunity the data group could pursue, what would you want that to be?
