# Supplementary material for: Data Access and Usage Practices Across a Cohort of Researchers at a Large Tertiary Pediatric Hospital: Qualitative Survey Study
Source: JMIR Med Inform. 2018 May 14;6(2):e32. doi: 10.2196/medinform.8724 (PMC5972187; doi:10.2196/medinform.8724)
Supplement: Multimedia Appendix 2 [file medinform_v6i2e32_app2.pdf]

Appendix B - List of Available Datasets Provided to Investigators

Holder	Dataset/Data Holdings
PopData (Population Data BC)	**Internal**<br>*Health*<br>- MSP (Medical Services Plan)<br>- PharmaCare<br>- Hospital Separations<br>- Home & Community Care<br>- Mental Health<br>- BC Cancer Registry<br>- Perinatal Data Registry<br>*Population and Demographic*<br>- BC Generations Project<br>- Permanent Residents<br>- MSP & Premium Billing<br>- Vital Statistics<br>o Births<br>o Stillbirths<br>o Marriage<br>o Deaths<br>- Income Band<br>*Occupational*<br>- WorkSafe BC Claims & Firm Level Files<br>- Integrated Cadastral Information Society (ICIS)<br>*Childhood*<br>- Early Development Instrument (HELP)<br>- Middle Years Development Instrument<br>- Provincial Standard Testing<br>**External**<br>- Vancouver Costal Health Authority<br>- BC MS Society<br>- BC Ministry of Health PharmaNet<br>- BC Transplant Society<br>- BC Centre for Excellence in HIV/AIDS<br>- College of Physicians and Surgeons of BC<br>- BC Centre for Disease Control<br>- Trauma Services BC<br>- BC Provincial Renal Agency<br>- BC Forest Safety Council

	- Cardiac Services BC<br>- BC Coroners Services<br>- LifeLabs
CIHI (Canadian Institute of Health Information)	- Discharge Abstract Database (DAD)<br>- National Ambulatory Care Reporting System (NACRS)<br>- Hospital Morbidity Database (HMDB)<br>- National Rehabilitation Reporting System (NRS)<br>- Continuing Care Reporting System (CCRS)<br>- Home Care Reporting System (HCRS)<br>- Hospital Mental Health Database (HMHDB)<br>- Ontario Mental Health Reporting System (OMHRS)<br>- Canadian Organ Replacement Register (CORR)<br>- National Trauma Registry (NTR)<br>- Ontario Trauma Registry (OTR)<br>- Canadian Joint Replacement Registry (CJRR)<br>- Medical Imaging Technology Database (MITDB)<br>- Canadian Multiple Sclerosis Monitoring System (CMSMS)<br>- National Prescription Drug Utilization Information System Database (NPDUIS)<br>- National System for Incident Reporting (NSIR)<br>- Canadian Patient Experiences Reporting System (CPERS)<br>- National Physician Database (NPDB)<br>- Scott's Medical Database (SMDB)<br>- Health Workforce Database (HWDB)<br>- National Health Expenditure Database (NHEX)<br>- Canadian MIS Database (CMDB)<br>- Canadian Patient Cost Database (CPCD)<br>- Organisation for Economic Co-operation and Development Health Database–Canadian Segment (OECD)<br>- Commonwealth Fund Survey(CMWF)<br>- Wait Times
HELP (Human Early Learning Partnership)	- Early Development Instrument<br>- Middle Years Development Instrument
Perinatal Services BC	- BC Perinatal Data Registry
PMR (Performance Measurement and Reporting)	- Personal Identifiers (C&W)<br>- Physician Encounters Database (BC Children's Out Patient Clinics)<br>- Nurse Practitioner Reporting Database<br>- BC Perinatal Data Registry<br>- Cerner

	- PHSA Corporate Statistics Database<br>- Discharge Abstract Database (DAD)<br>- Mental Health Database<br>- National Ambulatory Care Reporting System (NACRS)<br>- Operating Room Scheduling and Operating System (ORSOS)<br>- Workload Measurement System Database
Canadian Neonatal Network/Canadian Neonatal Follow-Up Network	- Sociodemographic information and post-NICU discharge health utilization<br>- Growth and physical examination<br>- CP and Gross Motor Function Classification System<br>- Bayley Scales of Infant and Toddler Development (3rd edition)<br>- Health Status Classification Pre-School (HSCS-PS)<br>- Ages and Stages Questionnaire<br>- Behaviour Rating Inventory of Executive Function-Preschool (BRIEF-P)
Clinical Databases	- *NSQIP*<br>- *VPS (Virtual Pediatric Intensive Care Unit Performance System)*<br>- *Vital Signs Database (BCCH)*
BioBanks	- BCCH<br>- BCCA [BC Cancer Agency Tumour Tissue Repository (TTR)]<br>- Canadian Tissue Repository Network<br>- Ontario Tumour Bank
Edudata (Ministry of Education)	- Course Level Data (e.g. course mark, course type)<br>- Student Level Data (e.g. Gender, birthdate)<br>- School Level Data (e.g. school name, location)<br>- FSA Variables Data (e.g. scores, grade level)
Other	- CHIRPP<br>- Please specify: _______________
