# Supplementary material for: Data Access and Usage Practices Across a Cohort of Researchers at a Large Tertiary Pediatric Hospital: Qualitative Survey Study
Source: JMIR Med Inform. 2018 May 14;6(2):e32. doi: 10.2196/medinform.8724 (PMC5972187; doi:10.2196/medinform.8724)
Supplement: Multimedia Appendix 3 [file medinform_v6i2e32_app3.pdf]

Appendix C - Final Analysis Template

1. **Barriers**
   a. Technical
      i. Data quality and usability
      ii. Data duplication
      iii. Time
      iv. Ability to link data

   b. Economic
      i. Costs of data
      ii. Lack of manpower (resources)

   c. Environmental
      i. Awareness
      ii. Unclear processes

   d. Legal
      i. Ethics/privacy concerns

2. **Facilitators**
   a. Existing rapport
   b. Primary collection
   c. Using well established data sources

3. **Opportunities**
   a. Facilitation of data access
      i. Data navigator
      ii. Privacy advisor

   b. Single data system
      i. Migration to electronic

   c. Funding allocation
   d. Future projects
